# Adolescent Binge-Type Ethanol Exposure in Rats Mirrors Age-Related Cognitive Decline by Suppressing Cholinergic Tone and Hippocampal Neurogenesis

**DOI:** 10.3389/fnbeh.2021.772857

**Published:** 2021-10-22

**Authors:** Nicole L. Reitz, Polliana T. Nunes, Lisa M. Savage

**Affiliations:** Department of Psychology, Binghamton University – State University of New York, Binghamton, NY, United States

**Keywords:** ethanol, adolescence, aging, acetylcholine, neurogenesis

## Abstract

Heavy alcohol consumption followed by periods of abstinence (i.e., binge drinking) during adolescence is a concern for both acute and chronic health issues. Persistent brain damage after adolescent intermittent ethanol exposure in rodents, a model of binge drinking, includes reduced hippocampal neurogenesis and a loss of neurons in the basal forebrain that express the cholinergic phenotype. The circuit formed between those regions, the septohippocampal pathway, is critical for learning and memory. Furthermore, this circuit is also altered during the aging process. Thus, we examined whether pathology in septohippocampal circuit and impairments in spatial behaviors are amplified during aging following adolescent intermittent ethanol exposure. Female and male rats were exposed to intermittent intragastric gavage of water (control) or 20% ethanol (dose of 5 g/kg) for a 2 days on/off cycle from postnatal days 25–55. Either 2 (young adult) or 12–14 (middle-age) months post exposure, rats were tested on two spatial tasks: spontaneous alternation and novel object in place. Acetylcholine efflux was assessed in the hippocampus during both tasks. There was no adolescent ethanol-induced deficit on spontaneous alternation, but middle-aged male rats displayed lower alternation rates. Male rats exposed to ethanol during adolescence had blunted behavioral evoked acetylcholine during spontaneous alternation testing. All ethanol-exposed rats displayed suppression of the cholinergic neuronal phenotype. On the novel object in place task, regardless of sex, ethanol-exposed rats performed significantly worse than control-treated rats, and middle aged-rats, regardless of sex or ethanol exposure, were significantly impaired relative to young adult rats. These results indicate that male rats display earlier age-related cognitive impairment on a working memory task. Furthermore, male rats exposed to ethanol during adolescence have blunted behavior-evoked hippocampal acetylcholine efflux. In addition, middle-aged and ethanol-exposed rats, regardless of sex, are impaired at determining discrete spatial relationship between objects. This type of pattern separation impairment was associated with a loss of neurogenesis. Thus, binge-type adolescent ethanol exposure does affect the septohippocampal circuit, and can accelerate age-related cognitive impairment on select spatial tasks.

## Introduction

Adolescence is a critical period of life that is marked by heightened levels of hippocampal neurogenesis as well as changes in neurotransmitter receptors/transporters and cortical synaptic remodeling (Bayer et al., 1982; He and Crews, 2007; Curlik et al., 2014). Binge drinking, the rapid rise of blood alcohol to above 80 mg/L in less than 2-h on an episodic time frame, during such a critical developmental epoch can lead to brain pathologies that last well into adulthood (Spear, 2000; Hiller-Sturmhöfel and Spear, 2018). Neuropathological studies in humans with a history of heavy alcohol use during adolescence show alterations in cortical excitability and functional brain connectivity, as well as shrinkage of hippocampal, prefrontal cortical, and cerebellar volume (De Bellis et al., 2000; Nagel et al., 2005; Jacobus and Tapert, 2013).

Adolescent intermittent ethanol exposure (AIE) is one of many models (see Spear, 2020) replicating extreme binge-like drinking (over 200 mg/dl, see Nguyen-Louie et al., 2016) in rodents by oral gavage of ethanol on a 2 days on, 2-day off procedure throughout the peri-adolescent to advanced adolescent time period. Further, adolescents are more susceptible to alcohol-related brain damage than adults are, especially within the septohippocampal circuit (Nixon and Crews, 2004; Broadwater et al., 2014; Vetreno et al., 2014; Fernandez and Savage, 2017). Two of the major brain pathologies observed as a result of AIE are the reduction in basal forebrain neurons expressing the cholinergic phenotype and decreased levels of hippocampal neurogenesis (Crews et al., 2019).

The medial septum/diagonal band (MS/DB) is a major hub of cholinergic neurons in the forebrain that is responsible for sending projections to the hippocampus, including the dentate gyrus (Mesulam et al., 1983; Rye et al., 1984). The septohippocampal circuit is critical to learning and memory, so alterations in this pathway can lead to significant behavioral dysfunction. Following AIE, there is a 25–30% suppression of cholinergic neurons in the MS/DB (Ehlers et al., 2011; Vetreno et al., 2014; Fernandez and Savage, 2017).

Decreased cholinergic tone within the septohippocampal circuit likely contributes to decreased hippocampal neurogenesis (Brooks et al., 1999; Cooper-Kuhn et al., 2004; Asrican et al., 2016; Kirshenbaum et al., 2018). Drugs that increase acetylcholine (ACh) levels have been shown to also enhance hippocampal neurogenesis (Kotani et al., 2006). Furthermore, the nicotinic α7 receptor partially regulates the survival of newly born adult neurons (Kita et al., 2014; Otto and Yakel, 2019). The dorsal hippocampus, relative to the ventral hippocampus, receives greater innervation from the MS/DB (Niewiadomska et al., 2009; Ballinger et al., 2016) and has a higher expression of doublecortin, a neuroprogenitor microtubule-associated protein expressed specifically by immature neurons (Jinno, 2010; Piatti et al., 2011). Following an AIE exposure, rats exhibit a 48 and 46% reduction of neurogenesis in the dorsal and ventral hippocampus, respectively (Vetreno and Crews, 2015). The dorsal hippocampus is largely involved in temporal cognition and spatial learning and memory, so neurogenesis within this region contributes to encoding and storage of memories (Moser and Moser, 1999; Kheirbek et al., 2013). It is argued that dorsal hippocampal adult-born neurons are recruited during spatial learning to encode detailed spatial and temporal information (Aimone et al., 2006; Kee et al., 2007; Rangel et al., 2014). As these new neurons pass through a critical period and integrate into pre-existing circuitry, they essentially take their encoded spatial and temporal information with them. This allows for the continuous stream of immature adult-born neurons to encode other distinct information about time and space, limiting the opportunity for overlap in similar spatial and temporal memories. In other words, dentate gyrus neurogenesis is strongly implicated in an organism’s ability to utilize pattern separation (Wiskott et al., 2006; Leutgeb et al., 2007; McHugh et al., 2007; Clelland et al., 2009; Sahay et al., 2011).

Exposure to binge-like ethanol consumption leads to a decrease in neurogenesis, evidenced by a decrease in the number of cells expressing doublecortin (Broadwater et al., 2014; Vetreno and Crews, 2015; Macht et al., 2020). The reductions in doublecortin immunopositive cells following AIE not only lasted into adulthood, but rapidly declined even further 7 months post-AIE (Vetreno and Crews, 2015). There is limited information about how adolescent alcohol exposure alters the trajectory of brain aging. Hippocampal neurogenesis rapidly declines with aging: By 9–12 months of age there is an 85–90% reduction in neurogenesis that is reduced even further by 20 months (Bondolfi et al., 2004; Ben Abdallah et al., 2010; Gil-Mohapel et al., 2013; Morel et al., 2015). Not only is neurogenesis declining, but neural progenitor cells also migrate much slower and much shorter distances as rodents age (Shuboni-Mulligan et al., 2019). Decreases in hippocampal neurogenesis due to aging likely contributes to age-related cognitive decline (Fischer et al., 1989; Gage et al., 1989).

Furthermore, there is also a loss of cholinergic neurons as the brain ages (Gu et al., 2000; Schliebs and Arendt, 2011). Pathologies associated with aging typically result in the loss of cholinergic phenotype, where natural aging results in loss of function due to dendritic, synaptic, and axonal degeneration (Schliebs and Arendt, 2011). A loss of cholinergic tone has been associated with memory impairments in aged populations (Disterhoft et al., 1999).

It appears that a dysfunctional septohippocampal circuit caused by AIE creates a vulnerability to the aging process, making an individual more susceptible to age-related cognitive decline earlier in life. Therefore, the effect AIE has on the septohippocampal circuit during aging needs to be investigated. Despite the evidence surrounding the loss of forebrain cholinergic phenotype and the decrease in hippocampal neurogenesis following AIE, hippocampal-dependent spatial behavioral deficits are not consistently observed (Vetreno and Crews, 2012; Swartzwelder et al., 2015; Fernandez and Savage, 2017; Fernandez et al., 2017). However, the AIE induced changes in the septohippocampal circuit may create a vulnerable state as subjects age. We explored the hypothesis that AIE-induced changes in the cholinergic system and hippocampal neurogenesis will be a significant contributor to unsuccessful brain aging, reflected by an early life exacerbation of age-related memory impairments. We included two spatial tasks with varying demands on memory and measured cholinergic tone during behavior. In addition, to gain insight into brain-behavioral relationships we analyzed cholinergic pathology and suppression of hippocampal neurogenesis.

## Materials and Methods

### Subjects

Subjects were male (*N* = 26) and female (*N* = 25) rats bred at Binghamton University and ranged from postnatal day (P) 25–27 at the start of treatment (see below). Only one rat per sex per litter was randomly assigned to a given treatment. Rats were pair-housed post weaning and given free access to food and water. All rats were housed in a temperature-controlled colony room that maintained a 12-h light/dark cycle from 7:00 AM to 7:00 PM. Rats were enriched with wood chew blocks and housed in standard cedar bedding. All procedures and housing were in accordance with the Institutional Animal Care and Use Committee (IACUC) of Binghamton University.

### Adolescent Intermittent Ethanol Exposure

**FIGURE 1:**
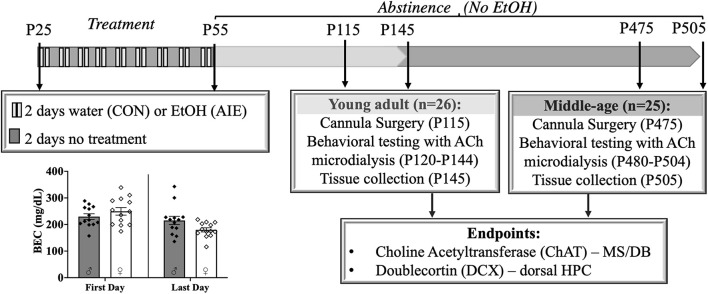
Experimental timeline. Male and female rats were exposed to a binge-type gavage ethanol exposure (5.0 g/kg 20% EtOH v/v, i.g) or control water exposure on a 2-day on/2-day off administration schedule from postnatal day (P) 25 to P55. Blood ethanol content (BEC) of each animal was measured 1 h after the first EtOH exposure (P25) and 1 h after the final exposure (P55). BECs did not significantly differ between the males and females. Upon completion of treatment, rats were further randomly split into two age relevant time points, Young- Adult and Middle-age, for cannula implantation surgery, behavioral testing and tissue collection. Choline Acetyltransferase (ChAT) positive cells in the medial septum/diagonal band (MS/DB) and doublecortin (DCX) positive cells in dorsal hippocampus (HPC) were counted to document pathology in the septohippocampal pathway.

Rats, both male and female, were either exposed to a binge-type forced ethanol exposure (AIE: *N* = 25) or were control exposure (CON: *N* = 26). Rats were exposed to 16 intragastric gavages of either 20% ethanol (EtOH) (*v/v*) or tap water, both of which were administered at a dose of 5 g/kg (see Figure 1). The exposure cycle followed a 2 days on/off cycle from P25–55. Tail blood samples were collected 1 h after the first exposure (P25) and 1 h after the final exposure (P55). All blood samples were processed using an AM1 Alcohol Analyzer (ANALOX Instruments, England) and the blood ethanol content (BEC) of each animal was recorded.

After completion of the ethanol or water exposure period, male and female rats were further randomly split into two time points for behavioral testing: Young Adult (4 months old; 2 months post-AIE) and Middle-age (14–16 months old; 12–14 months post-AIE).

### Cannula Implantation Surgery

Either 2-months or 12–14 months following AIE, all rats underwent surgery for a hippocampal cannula implantation. A mixture of ketamine (85 mg/kg; 4.25 mL), dexmedetomidine (0.04 mg/kg; 0.40 mL), and sterile saline (0.35 mL) was administered intraperitoneally at a dosage of 1 ml/kg for anesthetic purposes. Rats were placed in a stereotaxic apparatus (David Kopf Instruments, Tujunga, CA). Holes were drilled for the guide cannula (AP = −5.3 mm, ML = 5.1 mm, DV = −3.2 mm) and for the anchoring bone screws (placed arbitrarily near the cannula site). Guide cannula (8 mm; Synaptech Technology Inc., Marquette, MI, United States) were inserted in the hippocampus and fixed onto the skull using dental acrylic cement (Stoelting, Chicago, IL, United States). Carprofen (5 mg/kg) was administered prior to the surgery, 24 h following the surgery, and 48 h after the surgery for analgesic purposes. Rats were allowed 10 days of recovery before behavioral testing began.

### Behavioral Testing With *in vivo* Microdialysis

All rats were food restricted to 95% of their determined free-feeding weight and were handled for 5 days prior to testing. *In vivo* microdialysis was conducted during two behavioral tests, spontaneous alternation (SA) and novel object in place discrimination (NOP). Hippocampal microdialysis probes [2 mm membrane (SA); 4 mm (NOP) Synaptech Technology Inc.] were inserted into the cannula guide. The probes were attached to a microinfusion pump (CMA/400 pump) and an artificial cerebrospinal fluid solution (7.4 pH solution: 127.6 mM NaCl, 0.9 mM NaH_2_PO_4_, 2 mM Na_2_HPO_4_, 4 mM KCl, 1.3 mM CaCl_2_ dihydrate, 1.0 mM glucose, and 0.9 mM MgCl_2_) with 250 nM neostigmine hydrobromide (Sigma-Aldrich Corp.) was perfused continuously at a rate of 2.0 μL/min. Rats were habituated to the neostigmine perfusion in an opaque chamber for 60 min prior to behavioral testing.

Spontaneous alternation testing was performed using plus maze (105.5 cm × 14.4 cm × 15 cm) with clear exterior walls. The testing room had spatial cues adhered to the walls to allow the rats to navigate themselves through the maze. The spatial cues were the same for every rat. Following the habituation period, dialyzate was collected in 6-min samples for 18 min prior (baseline) and during testing (maze), and 24-min after testing (post-baseline). During testing, the rat was placed into the center of the plus maze and allowed to explore for 18 min. During this time, arm entries (defined as all four paws within an arm) were recorded and 6-min dialyzate samples were collected. One complete spontaneous alternation was defined as four consecutively unique arm entries. The rat’s performance was recorded as a percentage of alternation, or the ratio of actual alternations to possible alternations [total alternations/(total arm entries-3) × 100]. After testing was complete, the rat was returned to the habituation chamber for post-test microdialysis collection.

Novel object in place testing parameters were adapted from Cai et al. (2012). The NOP task was performed in an open-field arena (38.5 cm × 70 cm × 36 cm) with a black plexiglass floor and four white plexiglass walls. A video camera (Fujifilm, Tokyo, Japan) was fixed above the arena to record the behavior of the rat during testing. The objects were identical square-shaped blocks of wood (6.5 cm × 6.5 cm × 2.5 cm) with five holes in them, and they were attached to the bottom of the arena with Velcro adhesive to prevent the rats from moving them (see Figures 3A,B). The arena and objects were cleaned with diluted antibacterial solution between each trial to eliminate olfactory cues. Video recordings of the rat during exposure and test trials were analyzed by blind experimenters after all rats completed testing.

Prior to testing, rats underwent 2 days of habituation to the arena followed by 2 days of exposure to the objects. The total amount of seconds exploring each object was recorded for both exposure trials and for the test trial. A discrimination ratio was calculated for each animal: Seconds spent exploring the object in the novel location was divided by total time exploring both objects. An exploration ratio of 0.5 indicates equal amounts of time spent exploring the object in the novel location and the object in the familiar location. Exploration ratios significantly above 0.5 equate to more time being spent with the object in the novel location.

#### Habituation

Rats were placed into the open and empty arena for 10 min on two consecutive days. They were allowed to explore the area. Habituation was not recorded.

#### Encoding

The objects were placed in the arena beside each other, each approximately 11.1 cm × 15.28 cm from the nearest walls (see Figure 3A). Approximately 24 h following the second habituation period, rats were placed into the arena for 6 min on two consecutive days and allowed to explore the two objects. Exploration was recorded and analyzed both days to serve as a baseline for exploration of each object.

#### Retention Testing

One object remained in its original corner and the other was moved adjacent to the first object. The novel placement of the object was placed approximately 15.4 cm × 35.25 cm away from its nearest walls (see Figure 3B). Approximately 24 h after the second exposure trial, rats were allowed to explore the arena for 6 min. Behavior was recorded and analyzed to determine differences in the exploration time between the two objects. ACh efflux was assessed only during the retention testing phase: Following a 60-min habituation period, a 12-min baseline period prior to testing allowed for collection of microdialysis samples at 6-min intervals. This was followed by a 6-min collection during the retention phase. After retention testing, additional samples were collected at a 6-min interval.

### High-Performance Liquid Chromatography

All dialyzate samples were initially frozen and later unthawed to assay for ACh using high-performance liquid chromatography (HPLC) with electrochemical detection (Eicom, United States). Acetylcholine peaks were quantified by comparison to peak heights of standard solutions (100 nm, 20 nm, and 4 nm standards). Chromatographs obtained every 18 min/sample was analyzed using the software program Envision (provided by Eicom, United States). Minimal detection was 5 femtomoles of ACh.

### Tissue Preparation

The week following completion of behavioral testing, rats were euthanized with Fatal-Plus (Vortech Pharmaceuticals, Dearborn, MI, United States) and perfused (Masterflex Easy-Load Console Drive; 7518-00; Cole Palmer Instrument Co., Vernon Hills, IL, United States) with ice cold phosphate-buffered saline and 4% paraformaldehyde in 0.1M phosphate buffer (pH = 7.2). Brains were post-fixed at 4° overnight in 4% PFA and then transferred to a 30% sucrose solution (in 0.1M PBS) at 4° until slicing. Using a sliding microtome (Sm2000r Leica Biosystems, Wetzlar, Germany), whole brains were sliced coronally at 40 μm. Tissue slices will be kept in 96-well plates filled with an antifreeze solution (62.8 mg NaH_2_PO_4_, 2.18 g Na_2_HPO_4_, 160 mL dH_2_O, 120 mL ethylene glycol, and 120 mL glycerol) at −20° until immunohistochemistry staining.

### Brightfield Immunohistochemistry

#### Choline Acetyltransferase

Every 5th coronal section (five total sections per rat) of the MS/DB per rat was processed for ChAT+ expression. Free-floating sections were washed using a standard 0.1M phosphate-buffered saline (PBS) (pH = 7.4). Sections were then quenched using a 0.6% hydrogen peroxide solution for 30 min, followed by another PBS wash. Tissue slices were then processed through an antigen retrieval protocol using a 10 mM tri-sodium citrate buffer (pH = 8.5) at 80°C for 30 min. After another wash in 0.1M PBS, tissue sections were then blocked in a solution consisting of 4% rabbit serum (S-5000; Vector Laboratories) and 0.1% Triton X-100 (X-100; Millipore Sigma; Burlington, MA, United States) in a 0.1M PBS solution. Tissue was incubated overnight at 4°C in ChAT primary antibody (AB144P; EMD Millipore; Billerica, MA, United States; 1:200 dilution) made in blocking solution. The next day, tissue sections were washed in 0.1M PBS and then incubated in a secondary antibody (biotinylated rabbit anti-goat IgG; BA-5000; Vector Laboratories; Burlingame, CA, United States; 1:200 dilution) made in blocking solution. Tissue was washed in 0.1M PBS and then incubated in an avidin/biotin complex (VECTASTAIN Elite ABC HRP Kit, Vector Laboratories) diluted in 0.1M PBS for 1 h. Sections were then washed in PBS and developed using SIGMA*FAST* 3,3′-Diaminobenzidine (DAB) tablets (D4418; Millipore Sigma; Burlington, MA, United States) dissolved in 0.1M PBS. Following the ChAT+ development, tissue was rinsed for a final time in 0.1M PBS. Sections were then mounted, and cover slipped using VectaMount permanent mounting medium (H-5000; Vector Laboratories).

#### Doublecortin

Every 6th coronal section (five total sections per rat) of the dorsal HPC per rat was processed for DCX+ expression in the granule cell layer of the dentate gyrus. Free-floating sections were washed using a standard 0.1M Tris–buffered saline (TBS) solution (pH = 7.4). Sections were then quenched using a 0.6% hydrogen peroxide solution for 30 min, followed by another TBS wash. Tissue slices were then processed through an antigen retrieval protocol using a 10 mM tri-sodium citrate buffer (pH = 8.5) at 80°C for 30 min. Following another wash in 0.1M TBS, tissue slices were blocked using a solution consisting of 4% rabbit serum (S-5000; Vector Laboratories) and 0.1% Triton X-100 (X-100; Millipore Sigma; Burlington, MA, United States) in 0.1M TBS. Tissue was incubated at 4°C overnight in DCX primary antibody (SC-8066; Santa Cruz Biotechnology; Dallas, TX, United States; 1:200 dilution) made in blocking solution. The next day, tissue was washed in 0.1 TBS and then incubated in a secondary antibody (biotinylated anti-goat IgG; BA-5000; Vector Laboratories; 1:200 dilution) made with blocking solution. Tissue was then washed in TBS and incubated in an avidin/biotin complex (VECTASTAIN Elite ABC HRP Kit (PK-6100), Vector Laboratories) for 1 h in a solution of 0.1M TBS. Tissue was then rinsed and developed using SIGMA*FAST* DAB tablets with Metal Enhancer (D0426; Millipore Sigma). After a final wash in 0.1M TBS, sections were mounted, and cover slipped used VectaMount permanent mounting medium (H-5000; Vector Laboratories).

### Manual Counts of Medial Septum/Diagonal Band and Dorsal Hippocampus Cells

**FIGURE 2:**
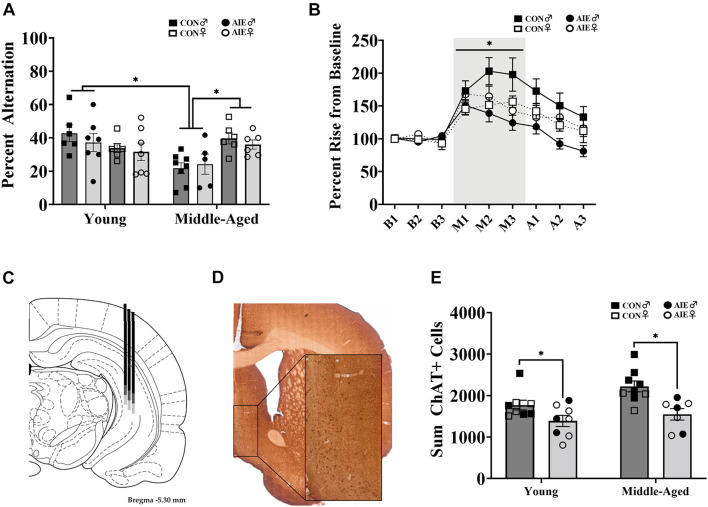
Sex differences on spatial working memory task. **(A)** Spontaneous alternation behavior. Young rats (AIE: Male = 7; Female = 7; CON: Male = 6; Female = 6) did not differ between sex or exposure (*p* = 0.15), but middle-aged males (CON = 8; AIE = 5) performed worse (^∗^*p* = 0.001) than middle-aged females (CON = 6; AIE = 6). Middle-aged males also performed worse than young adult males (*^∗^p* = 0.001), an effect not seen in females (*p* = 0.18). **(B)** Percent rise from baseline ACh levels in the hippocampus during spontaneous alternation testing. Data from young and middle-aged time points are combined in order to show Exposure × Sex × Phase interaction. Control male rats (*n* = 13) had a higher rise from baseline during testing than control females (*p = 0.005; *n* = 12), male AIE-exposed rats (*p* < 0.001; *n* = 12), and female AIE-exposed rats (*p* = 0.03, *n* = 12). **(C)** Examples of hippocampal microdialysis probe placement (AP = –5.3 mm, ML = 5.1 mm, DV = –5.2 mm) during spontaneous alternation is depicted by the solid dark gray line. Microdialysis probe placement (AP = –5.3 mm, ML = 5.1 mm, DV = –7.2 mm) during NOP is depicted by the lighter gray extension of the solid line. Image from Paxinos and Watson (2014). **(D)** Image of ChAT+ cells in the MS/DB (AP: 0.72 mm). Cells were quantified in one hemisphere on every 5th 40 μm section of the MS/DB. **(E)** AIE results in the suppression (27%) of cholinergic tone in the MS/DB (*p* < 0.001). Due to the quality of tissue, some samples were not included in cell quantification and sex differences were not able to be determined. For final ChAT+ counts: Young control males (*n* = 4), young control females (*n* = 5), young AIE males (*n* = 3), young AIE females (*n* = 5), middle-aged control males (*n* = 6), middle-aged control females (*n* = 3), middle-aged AIE males (*n* = 3), middle-aged AIE females (*n* = 4).

Images were captured using a SLIDEVIEW VS200 Research Slide Scanner (OLYMPUS Life Sciences; Waltham, MA, United States) with a 40X objective. Image analysis and blind manual cell counting (slides were coded to conceal subject, sex and treatment identity) was completed using the OlyVIA plugin for ImageJ. One hemisphere of each tissue section was quantified for immunopositive cells in the MS/DB (ChAT+; see Figure 2D) and dorsal HPC (DCX+; see Figure 4C), and the sum was doubled in order to represent both hemispheres in each tissue section. The total number of ChAT immunoreactive cells were quantified throughout the MS/DB and the total number of DCX immunoreactive cells were quantified throughout the dorsal dentate gyrus, according to the atlas of Paxinos and Watson (2014). The data are expressed as total (sum) cells throughout the MS/DB or dorsal HPC.

### Statistical Analyses

Analyses were performed in SPSS (IBM Corporation, Version 25). A univariate factorial ANOVA [Exposure (AIE, Control) × Age (Young, Middle-Aged) × Sex (Male, Female)] assessed spontaneous alternation and novel object in place measures, independently. *T*-tests were used to determine differences in NOP exploration ratios from a theoretical value of 0.50 (equal time spent with each object). A Pearson correlation assessed the linear relationship between NOP exploration ratios and total time (seconds) spent encoding objects, as well as the relationship between DCX+ cell counts and NOP exploration ratios. A repeated measures ANOVA, with Exposure, Age, and Sex as between subject factors, was used to analyze HPLC output. Error bars on graphs indicate the standard error of the mean.

## Results

### Binge-Like Blood Ethanol Concentrations

Adolescent intermittent ethanol exposed rats reached heavy binge-like levels of ethanol intoxication (∼239 mg/dl), well exceeding binge intoxication levels of at least 80 mg/dl (Spear, 2016). There was no difference in BECs between males (*M* = 229.80, *SD* = 38.51) and females [*M* = 249.61, *SD* = 51.24; *t*(24) = 1.11, *p* = 0.28]. BEC levels significantly decreased [*t*(25) = 26.84, *p* < 0.001] from the first (*M* = 239.70, *SD* = 45.54) to the last ethanol exposure (*M* = 197.57, *SD* = 47.15), but BECs consistently remained at high binge intoxication levels (see Figure 1). Body weights did not vary by exposure group (*p* = 0.86).

### Spontaneous Alternation

Although there were no main effects of Exposure, Age, or Sex [all *F*’s (1,7) < 3.59*;* all *p’*s > 0.07] on spontaneous alternation scores, there was a significant Age × Sex interaction [*F*(1,7) = 12.48, *p* = 0.001; see Figure 2A]. There were no differences in alternation rates between young males and young females (*p* = 0.23). Middle-aged males were impaired relative to young males (*p* = 0.001), but middle-aged females were not impaired relative to young females (*p* = 0.18). However, middle-aged males had lower spontaneous alternation scores than middle-aged females (*p* = 0.001). Thus, the sex-specific effect was that middle aged male rats were impaired, regardless of ethanol treatment.

There were no differences in activity, as a function of arm entries, due to Exposure [*F*(1,7) = 1.93, *p* = 0.17]. However, there was a main effect of Sex for arm entries: Male rats (*M* = 21.54, *SD* = 8.12) made fewer arm entries than females [*M* = 39.32, *SD* = 13.86; *F*(1,7) = 41.80, *p* < 0.001]. No interactions with Sex were found (*p* > 0.15). There was also a main effect of Age [*F*(1,7) = 16.08, *p* < 0.001]: Middle-aged rats (*M* = 24.60, *SD* = 11.93) made less arm entries than young rats (*M* = 35.69, *SD* = 14.56). To correct for this, we rescored spontaneous alternation data to include only the first 24 arm entries, as 24 arm entries was the mean for the middle-aged group. There were no main effects for Exposure, Age, or Sex [all *F*’s (1,7) < 2.49*;* all *p’*s > 0.12] on corrected spontaneous alternation scores. However, the interaction between Age × Sex remained [*F*(1,7) = 16.06, *p* < 0.001], with middle-aged male rats having lower alternation scores.

During spontaneous alternation testing, ACh efflux in the hippocampus (see Figure 2C) was measured in each rat. Analysis of ACh levels (in femtomoles) increased as a function of Phase (baseline, maze, after), with ACh efflux being greatest during spontaneous alternation maze testing, compared to baseline and post-maze levels [*F*(1,2) = 26.09, *p* < 0.001]. However, neither significant Sex (*p* = 0.76) nor Exposure (*p* = 0.47) effects were observed so femtomole data are not shown. When percent change from baseline was analyzed, there was a significant Exposure × Sex × Phase interaction (see Figure 2B) with control males having higher rise in ACh during testing than all other groups [*F*(1,1) = 3.32, *p* = 0.048]. Specifically, regardless of age, male AIE rats had lower behaviorally evoked ACh, compared to male control rats (*p* < 0.001). Although female control rats had lower behaviorally evoked ACh than male control rats (*p* = 0.005), regardless of age, they were not different from AIE female rats (*p* = 0.62).

### ChAT+ Cell Counts

AIE-exposed rats had a significant reduction (27%) in the total count of ChAT+ cells in the MS/DB compared to water control rats, regardless of age [*F*(1,28) = 16.27, *p* < 0.001; See Figures 2D,E]. There was no interaction between age and exposure (*p* = 0.28). Due to issues with tissue integrity in select subjects (tissue from 18/51 subjects was not included), we no longer had enough subjects to reliable determine sex as a factor. Subject numbers as a function of Exposure and Age are stated in the figure captions.

### Novel Object in Place

**FIGURE 3:**
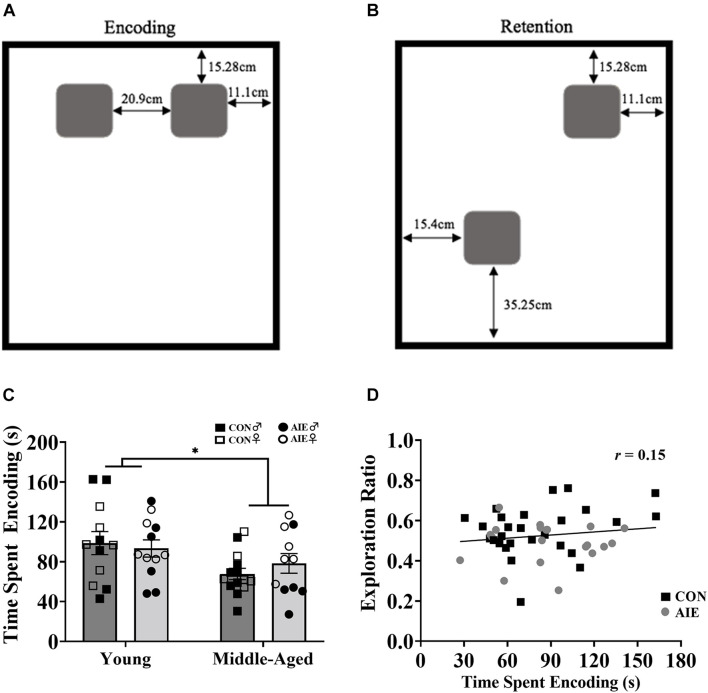
Rats underwent two encoding sessions (6 min each) followed by one retention test (6 min). **(A)** Set up of identical wooden blocks during the encoding phase of NOP. **(B)** Set up of identical wooden blocks during the retention testing phase of NOP. One object was moved to a novel location. **(C)** Exploration time during the encoding phase of NOP. Middle-aged rats, regardless of ethanol exposure, spent less time exploring the objects compared to young rats (^∗^*p* = 0.006). **(D)** Despite middle-aged rats spending less time with objects during the encoding phase and performing worse on the NOP task, encoding exposure time does not reliably predict retention test exploration ratios (*r* = 0.15, *p* = 0.318). For NOP results: Young control males (*n* = 6), young control females (*n* = 6), young AIE males (*n* = 6), young AIE females (*n* = 6), middle-aged control males (*n* = 8), middle-aged control females (*n* = 6), middle-aged AIE males (*n* = 5), middle-aged AIE females (*n* = 6).

During the encoding sessions middle-aged rats, regardless of Exposure or Sex, spent less time exploring the objects/locations, compared to young rats [main effect of age, *F*(1,7) = 8.88, *p* = 0.006; see Figure 3C]. However, longer time spent encoding did not predict better discrimination ratios during the retention session, as determined by a Pearson correlation analysis (Figure 3D). There was no main effect of Sex (*p* = 0.09) on time spent encoding.

**FIGURE 4:**
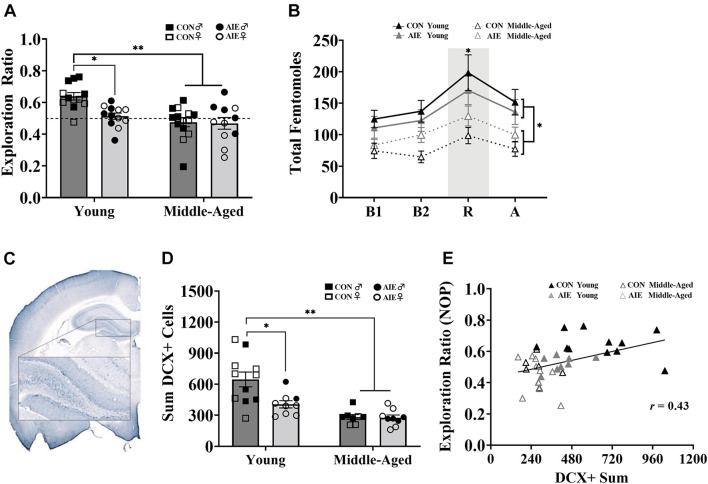
AIE and age similarly affect retention of object placement and neurogenesis in the dentate gyrus. **(A)** Young AIE exposed rats (Male = 6; Female = 6) had lower exploration ratios (^∗^*p* < 0.05) than young control rats (Male = 6; Female = 6). Overall, middle-aged rats (AIE: Male = 5; Female = 6; CON: Male = 8; Female = 6) had lower exploration ratios than young rats (^∗∗^*p* < 0.001). There were no effects of sex on NOP performance (*p* = 0.15). An exploration ratio of 0.5 (dashed line) indicates equal amounts of time spent with the object in the novel location and the object in the familiar locations. **(B)** Middle-aged rats, regardless of sex, had suppressed total femtomole efflux of ACh during baseline [B1, B2; (^∗^*p* < 0.001)], NOP retention (R) testing (^∗^*p* = 0.006) and after **(A)** testing (^∗^*p* = 0.05). **(C)** Image of DCX+ cells in the granule cell layer of the dentate gyrus (AP: –3.24 mm). Cells were quantified in one hemisphere on every 6th 40 μm section of the dorsal HPC. **(D)** Young AIE exposed rats had fewer DCX+ cells in the granule cell layer of the dentate gyrus compared to age-matched controls (^∗^*p* < 0.05). Overall, middle-aged rats had fewer DCX+ cells in the dentate gyrus compared to young water control rats (^∗∗^*p* < 0.001). Due to the quality of tissue, some samples were not included in cell quantification and sex differences were not able to be determined. For final DCX+ counts: Young control males (*n* = 4), young control females (*n* = 7), young AIE males (*n* = 2), young AIE females (*n* = 7), middle-aged control males (*n* = 5), middle-aged control females (*n* = 3), middle-aged AIE males (*n* = 4), middle-aged AIE females (*n* = 5). **(E)** Levels of neurogenesis in the dentate gyrus of the dorsal HPC correlate with performance on a pattern separation task. Exploration ratios on the NOP task positively correlated with sum values of DCX+ cells (*r* = 0.43, *p* = 0.007).

Assessment of the retention of object locations revealed a main effect of Exposure (Figure 4A). AIE exposed rats had a lower NOP exploration ratio during the retention phase than control rats, an effect driven by the young adult animals [*F*(1,7) = 5.64, *p* = 0.02]. Additionally, young AIE rats displayed chance level exploration ratios during the retention phase (*t* = 1.33, *p* = 0.22), whereas young control rats displayed a higher than chance level exploration ratio (*t* = 7.51, *p* < 0.001). There was also a main effect of Age: Overall, middle-aged rats had lower NOP exploration ratios than young rats, [*F*(1,7) = 14.83, *p* < 0.001]. Both AIE exposed and middle-age control rats displayed chance level exploration ratios during retention testing (both *p*’s > 0.31). There was an Age × Exposure interaction [*F*(1,7) = 5.58, *p* = 0.02]: Young AIE rats had a lower NOP exploration ratio than young control rats (*p* = 0.001), but middle-aged control rats and middle-aged AIE rats had similar NOP exploration ratios (*p* = 0.89). Young AIE rats did not differ from middle-aged AIE rats on exploration ratios (*p* = 0.30). Significant sex interactions were not observed on the NOP task (most *p*’s > 0.61). However, a non-significant trend (*p* = 0.052) existed for females, regardless of age or exposure, to have slightly lower exploration ratios than males.

During NOP retention testing, ACh efflux in the hippocampus was measured in each rat. Analysis of total femtomoles indicated a main effect of phase, with ACh efflux being greatest during object-location exploration compared to baseline (pre-test) and post-test levels, [*F*(1,3) = 25.00, *p* < 0.001]. There was also a main effect of Age: Middle-aged rats had suppressed hippocampal ACh efflux during object-location exploration compared to young rats, [*F*(1,1) = 8.67, *p* = 0.006; Figure 4B]. There were no effects of Exposure or Sex on total femtomoles of ACh during NOP testing (both *p*’s > 0.64). However, ACh efflux in male rats was more affected due to aging compared to female rats (*p* = 0.004).

### DCX+ Cell Counts

AIE resulted in a significant decrease (37%) in the total count of DCX+ cells in the dentate gyrus compared to water gavage controls [*F*(1,33) = 6.35, *p* = 0.017], an effect driven primarily by young adult rats (see Figure 4D). There was also a significant effect of Age: Although middle-aged control rats and middle-aged AIE exposed rats had a similar number of DCX+ cells (*p* = 0.83), both were reduced (by 56 and 57%, respectively) compared to young control rats [main effect of age, *F*(1,33) = 24.95, *p* < 0.001]. Furthermore, young adult AIE rats had a higher number of DCX+ cells than middle-aged AIE rats (*p* = 0.009). Due to issues with tissue integrity in select subjects (tissue from 15/51 subjects was not included), we no longer had enough subjects to reliable determine sex as a factor. Subject numbers as a function of Exposure and Age are stated in the figure caption.

As neurogenesis in the dentate gyrus is critical to pattern separation (Wiskott et al., 2006; Leutgeb et al., 2007; McHugh et al., 2007; Clelland et al., 2009; Sahay et al., 2011), a correlation was run to determine if NOP performance was associated with markers of neurogenesis. Higher levels of neurogenesis, as determined by DCX+ cells in the dorsal HPC, was positively correlated with higher retention ratios on the NOP task (*r* = 0.43, *p* = 0.007; see Figure 4E).

## Discussion

Given that adolescence is a highly critical period of brain development, critical events such as stressors or drug exposure can have long-lasting consequences (Spear and Swartzwelder, 2014; Spear, 2016, 2018), which may impact successful aging (Deak and Savage, 2019). The septohippocampal circuit is especially vulnerable to the effects of AIE, as previous research has found a significant suppression in basal forebrain neurons expressing the cholinergic phenotype, as well as decreased levels of neurogenesis in the hippocampus (Nixon and Crews, 2004; Broadwater et al., 2014; Vetreno et al., 2014; Fernandez and Savage, 2017). This circuit is also vulnerable to aging, including cholinergic cell loss (Mesulam, 2012; Stanley and Fadel, 2012; Norvin et al., 2015) and loss of neurogenesis (Kuhn et al., 2018; Mosher and Schaffer, 2018). Thus, the septohippocampal circuit, and the behaviors associated with it, are a key place to examine the role of developmental ethanol exposure in the progression of age-related cognitive decline.

The key findings of this study are as follows: binge-ethanol exposure during adolescence mirrors age-related cognitive decline, as shown by impaired pattern separation on the NOP task, which was associated with the suppression of hippocampal neurogenesis. Spatial working memory was selectively impaired in middle-aged male rodents, but was not affected by AIE exposure. Acetylcholine efflux in the hippocampus was differently affected by sex, age, and ethanol exposure depending on the nature of hippocampal task demands when ACh is being measured. During spontaneous alternation, male control rats (regardless of age) have a higher ACh efflux than male rats exposed to AIE, as well as a higher ACh than female rats, which do not differ as a function of ethanol treatment. During NOP, however, young control rats (regardless of sex) have higher ACh peaks during the retention than any middle-aged rats.

Despite persistent evidence of neuroanatomical disruption to the septohippocampal pathway following adolescent ethanol exposure, the hippocampal-dependent *behavioral consequences* are not always observed. To elucidate this discrepancy, the present study used two hippocampal spatial-dependent tasks that vary in retention time and spatial detail. It was found that the NOP task was sensitive to AIE deficits, but spontaneous alternation was not.

Spontaneous alternation assesses spatial working memory and is largely dependent on the functionality of the dorsal hippocampus (Moser and Moser, 1999; Kheirbek et al., 2013). Beyond the medial septum/diagonal band, the dorsal hippocampus also receives input from the medial entorhinal cortex, which may support short-term spatial processing in a compensatory manner in response to a decrease in MS/DB cholinergic input (see Knierim, 2015). Memory load can influence the role of hippocampal ACh efflux in modulating behavior (Roland et al., 2014). The retention of more discrete spatial locations across a longer time scale, as seen in the NOP task, likely evokes pattern separation, which is highly is dependent on dentate gyrus neurogenesis (Wiskott et al., 2006; Leutgeb et al., 2007; McHugh et al., 2007; Clelland et al., 2009; Sahay et al., 2011). No compensatory alternative neural function may exist for processing such discrete spatial information, and therefore AIE-induced memory impairment is observed in the NOP task.

Hippocampal neurogenesis is critical to an organisms’ ability to encode and remember temporal and spatial information (Aimone et al., 2006; Kee et al., 2007; Rangel et al., 2014). Decreased levels of hippocampal neurogenesis seen in rats following AIE is likely to have an impact on dentate gyrus-dependent tasks (Zhao et al., 2006, 2008; Appleby and Wiskott, 2009; Bolz et al., 2015; Pan et al., 2019). Our results support this, as rats that underwent AIE had lower exploration ratios on an object location retention task, compared to control rats. We believe that the spatial parameters and the longer retention interval of the NOP task (24-h) made it more dependent on hippocampal neurogenesis, and therefore more disrupted by AIE, relative to the parameters of the short-term spatial spontaneous alternation task. This was supported by a reduction in DCX+ cells (37%) in the dentate gyrus of AIE-exposed rats when compared to age-matched water controls. A positive correlation between NOP exploration ratios and DCX+ cells (both in controls and AIE-exposed rats) further support the relationship between neurogenesis and pattern separation, and how AIE can strongly impact both. The loss of neurogenesis, assessed by DCX positive cell count, in middle-aged rats, was potentiated by AIE, supporting the age coupling hypothesis of alcohol-related brain damage in the hippocampus.

Hippocampal neurogenesis and ACh levels are complementary to one another (Kita et al., 2014; Otto and Yakel, 2019). Thus, the loss of neurogenesis and reductions in the basal forebrain cholinergic phenotype as a function of aging and developmental ethanol exposure may interact. Hippocampal ACh efflux is reduced as a result of aging (Wu et al., 1988; Chang and Gold, 2008; Stanley and Fadel, 2012). Age-related declines in ACh efflux have been found as early as 10 months old (Chang and Gold, 2008). Neurogenesis significantly decreases by 7 months in rats (Vetreno and Crews, 2015). Furthermore, hippocampal neurogenesis assists in maintaining the cholinergic projections of the septohippocampal circuit across the lifespan. A significant remodeling of the cholinergic septohippocampal projection occurred as a function of aging following hippocampal X-irradiation that significantly suppress neurogenesis, specifically there was a recruitment of ventrally projecting neurons for innervation of both the dorsal and ventral hilus across 5–7 months (Kirshenbaum et al., 2018). In addition, there is evidence of acetylcholine modulating neurogenesis (Veena et al., 2011). Lesioning of forebrain cholinergic inputs leads to a decrease in proliferation and differentiation of newly born hippocampal neurons (Mohapel et al., 2005), a deficit that is rescued by administration of the muscarinic agonist oxotremorine (Van Kampen and Eckman, 2010).

The effects of aging on cognition and brain function are variable. The trajectory of age-related cognitive decline is influenced by factors such as genetics, sex, and the environment, including drug exposure, which can interact to blunt or accelerate aging processes (McQuail et al., 2021). We found that AIE led to a blunting of behaviorally evoked ACh during the spontaneous alternation task selectively in male rats. Furthermore, by middle age, male rats began to show impairment on spontaneous alternation. On the NOP task, sex difference as a function of ethanol exposure or aging were not observed: Young-adult rats exposed to AIE had the same impairment as middle-aged rats, regardless of sex. There was not a synergistic effect between AIE and aging on retention of object locations, but this may be due to a floor effect. Advanced age or AIE may decrease discrete spatial memory to a dysfunctional level that is not further disrupted by additional brain damage (see Pitkin and Savage, 2004). There is some ecological validity to such a phenomenon as there is some level of functioning that is needed for an animal’s successful survival. Aging does not affect all systems equally and the interactions between aging and previous brain damage do not always lead to synergistic severe behavioral dysfunction (McQuail et al., 2021).

Furthermore, age-related cognitive decline is not absolute: Only a subset of aged rats have cognitive impairment as consequences of natural aging (Gage et al., 1984, 1989; Fischer et al., 1989). In the rodent, cognitive decline can begin to emerge in middle-age, but at this age cognitive performance is highly variable (Guidi et al., 2014). Natural aging in male rodents results in impaired spatial memory starting at 12 months of age (middle age; Bizon et al., 2009). Regardless of ethanol exposure, middle-aged rats performed worse than young rats on the NOP task, and middle-aged rats also had suppressed ACh efflux compared to controls, indicating that ACh efflux declines as rats age. By the time rats reach middle-age, their abilities to acquire episodic spatial memory are impaired (Guidi et al., 2014). Interestingly, middle-aged males performed worse on the spontaneous alternation task compared to middle-aged females. A recent longitudinal study (Febo et al., 2020) also found impairments in episodic spatial memory in the water maze selectively in middle-aged male rats. Thus, our data support a sex unique propensity for cognitive decline in middle age that is task dependent. Middle-aged male rats show impairment on both the spontaneous alternation task and the 24-h retention of object location. In contrast, middle age females are impaired on only the 24-h retention of object location. Interestingly, AIE rats regardless of sex are also impaired on the 24-h retention of object location. As stated earlier, by middle-age, there are no additive AIE-induced impairments on the NOP task. This is due to control middle-aged rats already performing at chance levels on objection location memory. It appears that pattern separation is affected so significantly by aging alone that any further impairments from early life ethanol exposure are not observable. Furthermore, male AIE rats, regardless of age, have blunted hippocampal ACh efflux while engaging in spontaneous alternation. Thus, developmental ethanol exposure, sex, advancing age and task demands influence cognitive dysfunction.

Estrous cycles were not monitored throughout the present study, so we cannot make any claims about whether neurogenesis levels, ACh levels, or spatial memory varied as a function of cycle as previously seen (Galea et al., 1995; Warren and Juraska, 1997; Galea and McEwen, 1999). However, in a previous study (Vedder and Savage, 2017) we found that estrus cycle did not affect spontaneous alternation behavior in young rats. Furthermore, between 6- and 18-months female rodents exhibit periods of persistent low levels of estrus (Lu et al., 1979; Nelson et al., 1981; vom Saal et al., 1994), and no longer cycle. Variability was not different as a function of sex on behavioral and brain measures.

In summary, binge-type ethanol exposure during adolescence does appear to resemble advancing age in both loss of cholinergic neuronal phenotype and impaired memory for discrete spatial locations. Furthermore, there are some sex differences in response to adolescence binge-type ethanol exposure and aging. Specifically, on the spontaneous alternation task, middle aged males show behavioral impairment, while AIE-treated male exhibit blunted ACh efflux while engaging in spontaneous alternation behavior. In contrast, no sex differences were seen in the retention of a discrete spatial locations, but such performance was impaired in rats exposed to binge type ethanol during adolescence and in middle age non-ethanol exposed rats. Pathology in the septohippocampal pathways was evident following adolescent ethanol exposure and aging. There was a loss of the cholinergic phenotype only following adolescent ethanol exposure. We did not see an overall loss of the cholinergic phenotype in middle-aged rats compared to young adults. This is not surprising given that other studies have not observed a reduction in the cholinergic phenotype until advanced age (20–24 months; Martínez-Serrano and Björklund, 1998; Pitkin and Savage, 2004). Sex dependent differences in cholinergic cell loss was not observed after adolescent ethanol exposure, but the sample size may have been too limited to detect sex differences.

In contrast, reductions in neurogenesis, as determined by doublecortin staining, were observed in rats exposed to ethanol as adolescents and in middle aged rats. Furthermore, the greatest decline in neurogenesis was seen in middle aged rats that were exposed to ethanol as adolescents. Thus, hippocampal neurogenesis is sensitive to the synergistic interaction between ethanol exposure and aging. Aging is a variable state with several factors influencing the trajectory into dementia. Exposure to ethanol, even during development– including the adolescent period, can mimic the behavioral effects of advanced aging and can lead to greater septohippocampal pathology.

## Data Availability Statement

The raw data supporting the conclusions of this article will be made available by the authors, without undue reservation.

## Ethics Statement

The animal study was reviewed and approved by the Binghamton University IACUC.

## Author Contributions

LS and NR designed the research. PN and NR performed the research. NR analyzed the data. LS, PN, and NR wrote the manuscript. All authors contributed to the article and approved the submitted version.

## Conflict of Interest

The authors declare that the research was conducted in the absence of any commercial or financial relationships that could be construed as a potential conflict of interest.

## Publisher’s Note

All claims expressed in this article are solely those of the authors and do not necessarily represent those of their affiliated organizations, or those of the publisher, the editors and the reviewers. Any product that may be evaluated in this article, or claim that may be made by its manufacturer, is not guaranteed or endorsed by the publisher.

## Abbreviations

ACh: acetylcholine
AIE: adolescent intermittent ethanol exposure
ChAT: choline acetyltransferase
HPC: hippocampus
MS/DB: medial septum/diagonal band
NOP: novel object in place
NGF: nerve growth factor.
