# Supplementary material for: Adolescent Binge-Type Ethanol Exposure in Rats Mirrors Age-Related Cognitive Decline by Suppressing Cholinergic Tone and Hippocampal Neurogenesis
Source: Front Behav Neurosci. 2021 Oct 22;15:772857. doi: 10.3389/fnbeh.2021.772857 (PMC8569390; doi:10.3389/fnbeh.2021.772857)
Supplement: Supplementary file 1 [file Image_1.pdf]

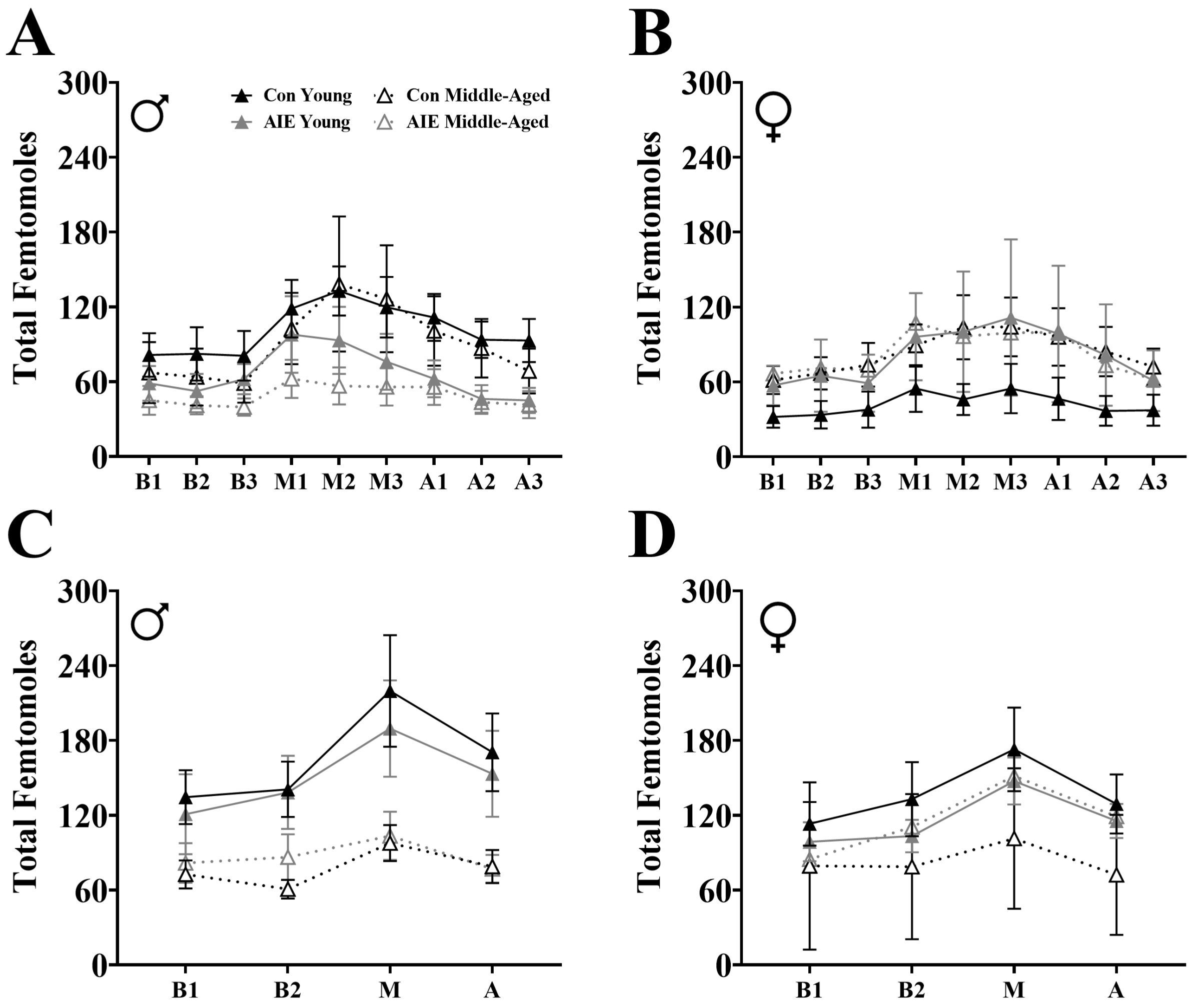

Supplementary Figure 1. Total femtomoles of ACh efflux before (baseline: B1, B2, B3), during (M1, M2, M3), and after (resting baseline: A1, A2, A3) testing on spontaneous alternation– in males. *B*) Total femtomoles of ACh before, during, and after testing on spontaneous alternation– in females. *C*) Total femtomoles of ACh efflux before (baseline: B1, B2), during (M), and after (resting baseline: A) testing on the novel object in place task– in males. *D*) Total femtomoles of ACh efflux before, during, and after testing on the novel object in place task – in females.
